# Use of video surveillance to measure the influences of habitat management and landscape composition on pollinator visitation and pollen deposition in pumpkin (*Cucurbita pepo*) agroecosystems

**DOI:** 10.7717/peerj.1342

**Published:** 2015-11-05

**Authors:** Benjamin W. Phillips, Mary M. Gardiner

**Affiliations:** Department of Entomology, The Ohio State University—Ohio Agricultural Research and Development Center, Wooster, OH, United States; 1 Current affiliation: Michigan State University, Saginaw, MI, United States

**Keywords:** Pollination services, Landscape, Pumpkin, *Peponapis pruinosa*, Bumble bee, *Apis mellifera*, Habitat managment, Floral strips

## Abstract

Pumpkin (*Cucurbita pepo*) production relies on insect-mediated pollination, which is provided by managed and wild pollinators. The goals of this study were to measure the visitation frequency, longevity and temporal activity patterns of pumpkin pollinators and to determine if local habitat management and landscape composition affected this pollination service. We used video surveillance to monitor bee acitivty within male and female pumpkin flowers in 2011 and 2012 across a pollination window of 0600–1200 h. We also quantified the amount of pollen deposited in female flowers across this time period. In 2011, *A. mellifera* made significantly more floral visits than other bees, and in 2012 *Bombus spp*. was the dominant pumpkin pollinator. We found variation in visitation among male and female pumpkin flowers, with *A. mellifera* visiting female flowers more often and spending longer per visit within them than male flowers in both 2011 and 2012. The squash bee *P. pruinosa* visited male flowers more frequently in 2012, but individuals spent equal time in both flower sexes. We did not find variation in the timing of flower visitation among species across the observed pollination window. In both 2011 and 2012 we found that the majority of pollen deposition occurred within the first two hours (0600–0800 h) of observation; there was no difference between the pollen deposited during this two-hour period and full pollination window (0600–1200 h). Local additions of sweet alyssum floral strips or a field buffer strip of native wildflowers did not have an effect on the foraging activity of bees or pollen deposition. However, semi-natural and urban habitats in the surrounding landscape were positively correlated with the frequency of flower visitation by wild pollinators and the amount of pollen deposited within female flowers.

## Introduction

Worldwide, 35% of the global food supply is highly reliant on animal-mediated pollination services (Klein et al., 2007; Nicholls & Altieri, 2013). In the United States alone, pollinators account for 40 billion USD per year in fruit, fiber, vegetable and legume crops (Pimentel et al., 1997), with an estimated 1.6–14.8 billion USD of that attributed to the honey bee, *Apis mellifera* L. (Hymenoptera: Apidae) (Southwick & Southwick, 1992; Morse & Calderone, 2000; Losey & Vaughan, 2006). Across the United States and Europe, severe declines in the supply of honey bees for crop pollination have occurred as a result of colony collapse disorder (Allen-Wardell et al., 1998; Aizen & Harder, 2009; Potts et al., 2010a; Potts et al., 2010b). Wild bee species also contribute significantly to pollination within many cropping systems (Stanghellini, Ambrose & Schultheis, 1998; Kremen, Williams & Thorp, 2002; Winfree et al., 2007; Garibaldi et al., 2013; Garibaldi et al., 2014). Unfortunately, several wild pollinator taxa—such as some bumble bee species—have also exhibited significant declines in richness and abundance, further threatening the continued supply of pollination services to agroecosystems (Goulson, Lye & Darvill, 2008; Cameron et al., 2011).

### Habitat management to support pollinators

Several potential drivers of population decline among pollinators have been identified, including pesticide use (Sanchez-Bayo & Goka, 2014; Rundlof et al., 2015), pathogen and parasite infection (Meeus et al., 2011; Blaker et al., 2014; Fuerst et al., 2014; Goulson et al., 2015; McMahon et al., 2015), exposure to heavy metals (Moran et al., 2012), climate change, land use change and fragmentation of pollinator habitat, or a combination of several factors (Potts et al., 2010a; Gonzalez-Varo et al., 2013; Rader et al., 2013; Vanbergen et al., 2013; Rands, 2014; Scheper et al., 2014; Goulson et al., 2015; Rollin et al., 2015). To address the impacts agricultural intensification may have on wild and managed bee populations, agri-environmental schemes have been designed to reestablish pollinator resources within agricultural landscapes (Haaland, Naisbit & Bersier, 2011; Rollin et al., 2015). Enhancing farmscape-scale heterogeneity through this form of habitat management has been demonstrated to increase pollinator richness by providing resources across time and space (Klein, 2011; Kennedy et al., 2013; Shackelford et al., 2013; Blaauw & Isaacs, 2014; Garibaldi et al., 2014). The flowering plants established in these plantings have been shown to be highly attractive to a diversity of beneficial insects, increasing fecundity, longevity, and the ecosystem services provided such as pollination and biological control (Baggen & Gurr, 1998; Johanowicz & Mitchell, 2000; Landis, Wratten & Gurr, 2000; Pontin et al., 2005; Lee, Andow & Heimpel, 2006; Pywell et al., 2006; Tuell et al., 2008). However, the addition of floral resources could in theory result in competition for pollinators with the target crop (Zhang et al., 2007). Thus, evaluation of the impacts of these strategies on foraging efficiency within specific agroecosystems is a necessary step towards incorporation of this conservation practice.

### Landscape context can influence the outcome of habitat management

When habitat management practices are incorporated into a farmscape, larger scale landscape composition and heterogeneity can influence the pool of beneficial species supplied to an established planting and the arthropod mediated ecosystem services they are able to support in nearby farm fields (Isaacs et al., 2009; Batáry et al., 2011; Concepción et al., 2012; Rodriguez-Saona, Blaauw & Isaacs, 2012; Tscharntke et al., 2012). In synthesis papers, Ricketts et al. (2008) and Garibaldi et al. (2011) found decreased stability and levels of pollination services provided by pollinator communities with increasing distance from natural areas. Kennedy et al. (2013) analyzed data from 39 studies focusing on 23 cropping systems and found that organically-managed cropping systems supported a greater abundance and richness of wild bees. Similar to previous reviews, they also documented that at landscape scales the proportion of high-quality natural habitat was positively related to bee abundance and richness. Further, landscape factors have been shown to mediate the impact of some agricultural inputs. For example, Park et al. (2015) found that pesticide impacts on wild bees in apple orchards were reduced in landscapes with high proprotions of natural habitat.

### Habitat management in cucurbit agroecosystems

As agricultural intensification threatens both natural pest control and pollination, habitat management strategies often target multiple key insect guilds (Campbell et al., 2012). The sustainability of pumpkin, *Cucurbita pepo* L. (Cucurbitales, Cucurbitaceae), production relies in part on biological control to suppress key pests. Being a monoecious crop, pumpkin is also dependent on insect-mediated pollination (Wien, 1997). Furthermore, pumpkin provides a unique study system to evaluate habitat management in sustaining pollination services because they are visited by managed (*A. mellifera*) and wild (*Bombus* spp.) social bees as well as a wild solitary specialist pollinator, *Peponapis pruinosa* (Say) (Hymenoptera: Apidae) (Hurd, Linsley & Whitaker, 1971; Hurd, Linsley & Michelbacher, 1974). Due to differences in foraging traits, greater pollinator richness within this system may lead to functional complementarity or synergy, thereby improving pollination efficiency (Bluthgen & Klein, 2011).

In a network of pumpkin farms across central and southern Ohio, we completed the following research objectives: (1) Use video surveillance to measure the relative contribution of pumpkin pollinator taxa to pollination services; (2) Determine if pollinators varied in their visitation frequency and visit longevity in male and female flowers; (3) Examine if temporal complementarity exists among flower visitation by pumpkin pollinators; and (4) Determine how habitat management and landscape composition influence pollination visitation and pollen deposition. This study was completed during the growing seasons of 2011 and 2012. In 2011, we measured how landscape composition influenced pollinator activity and pollination services within pumpkin crops. In 2012, we added habitat management as a variable and evaluated how the addition of floral strips of sweet alyssum *Lobularia maritima* (L.) (Brassicales: Brassicaceae) or a buffer strip of native perennial wildflowers and grasses—as well as the surrounding landscape—influenced pollinators and their function.

## Methods

### Study sites

**Figure 1:**
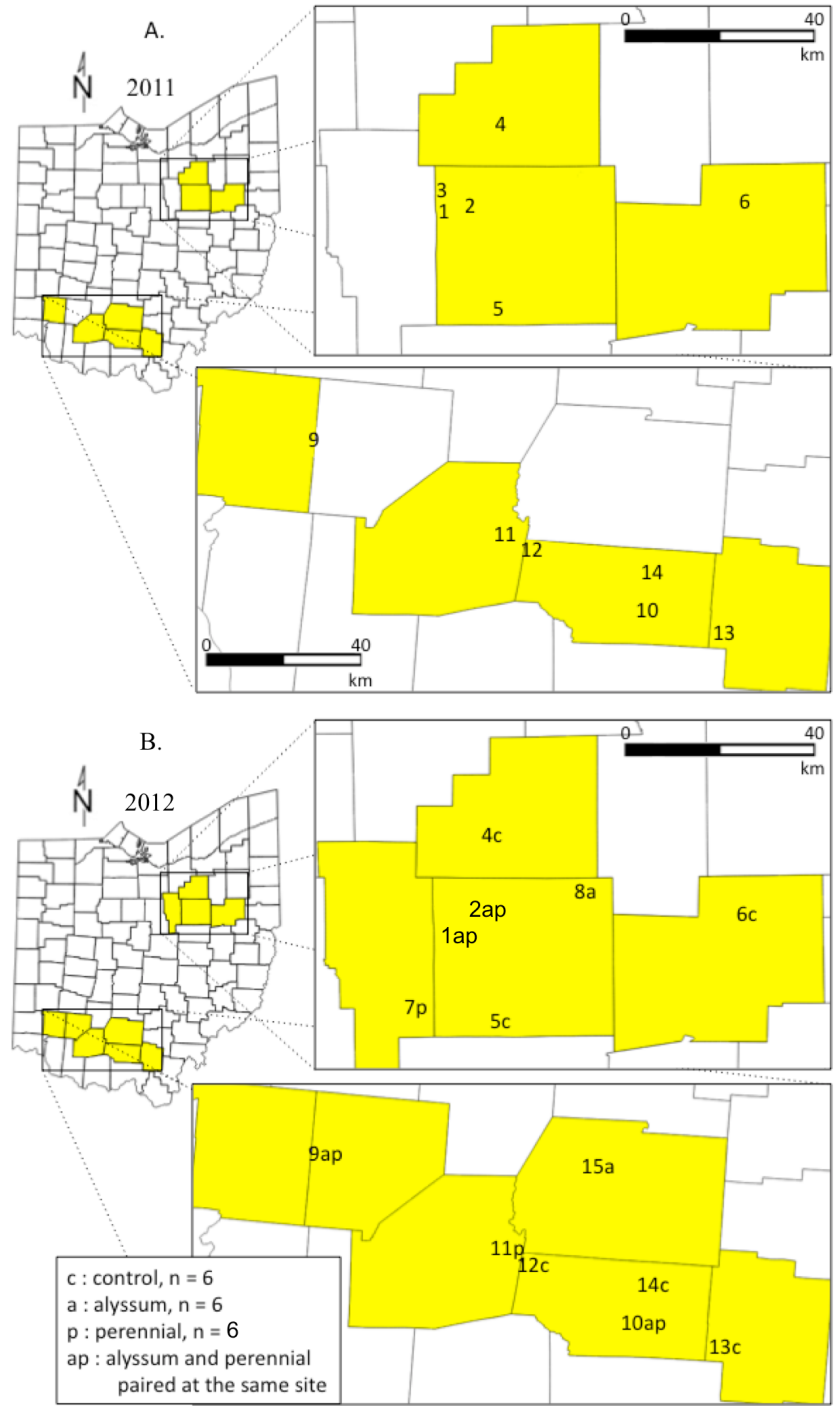
Pumpkin sites were located in growing regions in northern and southern Ohio. In 2011, we established 12 pumpkins sites on individual farms. We did not evaluate habitat management in 2011; each pumpkin site was adjacent to a grassy field border. In 2012, we added 6 additional pumpkin sites for a total of 18. Each site was assigned to one of three habitat management treatments: GRASS CONTROL (pumpkin plot adjacent to a 6 × 60 m grass area, mowed approximately once per month) (2) ALYSSUM (pumpkin plot planted between two 60 m rows of the non-native annual, *L. maritima*), and (3) PERENNIAL (pumpkin plot planted adjacent to a 6 × 60 m buffer of native perennial wildflowers). These sites were located on 15 farms. Each farm had one pumpkin site except for farms 1, 2, 9, and 10 where both one ALYSSUM and one PERENNIAL treatment site were established. The distances between these plots ranged from 51 m at site 10, to 570 m at site 9.

Ohio is the 2nd largest pumpkin-producing state in the United States, and two regions within Ohio were selected that represent major production areas (USDA-NASS, 2013). In 2011, 12 farms were included in our study; six in Wayne, Stark, and Medina counties in northern Ohio, and six in Jackson, Pike, Highland, and Warren counties in southern Ohio (Table 1, Fig. 1A). In 2012, 15 farms were included, with eight farms in northern Ohio and seven in southern Ohio (Table 1, Fig. 1B). The distance between the two closest farms was 4.25 km within a given year. Farms were chosen based on grower interest in participating and by assessment of the composition of habitats in the surrounding landscape. One to four *Apis mellifera* hives were located within each farm.

**Table 1:** Location of farms studied in 2011 and 2012. In 2011 we did not evaluate habitat management and each pumpkin site was adjacent to a grassy field boarder (*n* = 12). In 2012, each pumpkin site was assigned to one of three habitat management treatments: (1) GRASS CONTROL: four rows of pumpkin planted adjacent to a 6 × 60 m grass area, mowed approximately once per month; (2) ALYSSUM: four rows of pumpkin planted between two 60 m rows of *L. maritima*; and (3) PERENNIAL: pumpkin plots planted adjacent to a 6 × 60 m buffer of native perennials habitat management treatments. Farms 1, 2, 9, and 10 hosted two pumpkin sites in 2012.

	2011		2012		
Farm	Latitude	Longitude	Habitat treatment	Latitude	Longitude
1	40°54′37.94″N	82°6′35.06″W	Alyssum	40°54′9.69″N	82°6′44.11″W
1	–	–	Perennial	40°54′38.1″N	82°6′35.5″W
2	40°55′6.92″N	82°2′57.66″W	Perennial	40°54′58.95″N	82°2′48.14″W
2	–	–	Alyssum	40°55′5.27″N	82°2′38.15″W
3	40°56′25.06″N	82°6′58.21″W	–	–	–
4	41°5′2.65″N	81°57′1.51″W	Control	41°5′3.28″N	81°57′8.13″W
5	40°42′37.87″N	81°58′16.31″W	Control	40°42′23.5″N	81°57′56.45″W
6	40°55′17.93″N	81°18′33.26″W	Control	40°55′17.29″N	81°18′31.78″W
8	–	–	Alyssum	40°58′13.68″N	81°44′25.37″W
7	–	–	Perennial	40°44′12.27″N	82°11′48.86″W
9	39°26′5.63″N	83°59′26.59″W	Perennial	39°26′4.39″N	83°59′1.35″W
9	–	–	Alyssum	39°26′4.01″N	83°59′25.23″W
10	39°2′50.88″N	82°59′37.4″W	Perennial	39°2′49.35″N	82°59′38.15″W
10	–	–	Alyssum	39°2′50.88″N	82°59′37.4″W
11	39°13′13.41″N	83°25′36.81″W	Perennial	39°13′13.41″N	83°25′36.81″W
12	39°10′58.65″N	83°21′3.09″W	Control	39°10′55″N	83°21′11.37″W
13	38°59′29.9″N	82°46′4.54″W	Control	38°59′37.44″N	82°45′51.76″W
14	39°8′16.65″N	82°58′58.47″W	Control	39°8′11.46″N	82°58′59.39″W
15	–	–	Alyssum	39°24′41.94″N	83°9′27.33″W

In both years, data was collected from four 60 m rows of jack-o-lantern pumpkins (var. Gladiator), which were established between 10 June and 8 July. No insecticides were applied to the pumpkin plants throughout the study. We refer to each planting as a site. Each site was divided into four 15 m plots that each contained four rows of pumpkin, and all data were collected within these plots. In 2011, one pumpkin site was located per farm (*n* = 12). In 2012, a total of 18 pumpkin sites were established. Each farm had one site except for farms 1, 2, 9, and 10 where two sites were established (Fig. 1). This was the result of difficulty finding growers willing to host habitat management plantings. Farms 1, 2, 9, and 10 included both an ANNUAL and PERENNIAL treatment pumpkin site (see ‘Habitat management’). The distances between these sites ranged from 51 m at farm 10, to 570 m at farm 9.

### Habitat management

In 2012, pumpkin plots within the northern and southern regions were randomly assigned to one of three treatments: (1) GRASS CONTROL: four rows of pumpkin planted adjacent to a 6 × 60 m grass area, mowed approximately once per month; (2) ALYSSUM: four rows of pumpkin planted between two 60 m rows of *L. maritima*; and (3) PERENNIAL: pumpkin plots planted adjacent to a 6 × 60 m buffer of native perennials (Table 2).

**Table 2:** Native perennial floral insectaries consisting of 23 forbs and 2 grasses* were established in 6 × 60 m plots in 2010. The impact of these habitats on pollinator visitation frequency and pollination services was assessed in 2012. The seed mix was designed following Fiedler et al. (2007) and Tuell et al. (2008) to support the production of floral resources throughout the growing season.

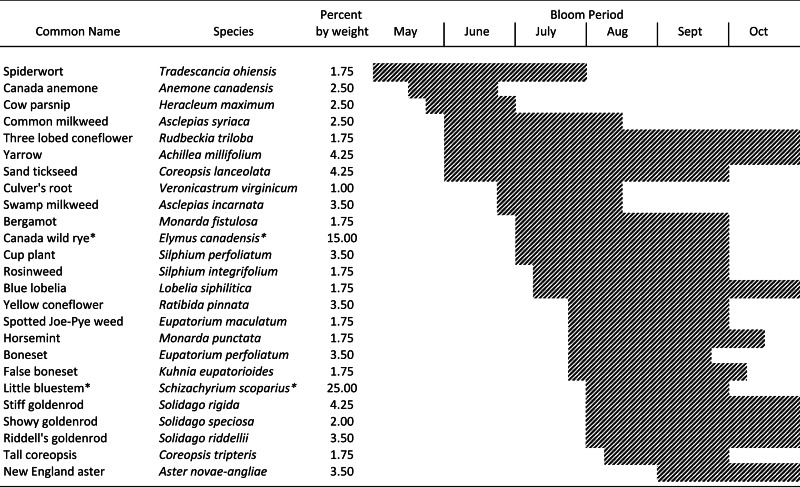

#### Establishing non-native annual floral insectaries

In 2012, we planted two rows of *L. maritima* adjacent to pumpkins at six sites in northern and southern Ohio. For this treatment, one row of *L. maritima* was established on either side of each four-row pumpkin planting. The *L. maritima* was started from seed in 72-cell plug trays in a greenhouse in early May and fertilized twice per week for two weeks. The plants were hardened off outside for an additional two weeks before being transplanted with a pottiputki planter (Stand ‘n Plant, Saltsburg, Pennsylvania, USA) between 7–14 June 2012. Plants were watered and Preen Garden Weed Preventer (Lebanon Seaboard Corp., Lebanon, Pennsylvania, USA) was applied. The transplants were watered via drip irrigation and hand containers (∼190 L) twice per week in the field through July.

#### Establishing native perennial floral insectaries

The perennial insectary was established in fall of 2010 to allow the plants time to establish prior to their evaluation in summer 2012. In October 2010, six farms were selected to establish a 6 × 60 m perennial floral insectary treatment of 23 native forbs and two grasses (Table 2). Each grower cleared the area with field cultivators and herbicide, and rolled the soil flat. We mixed the perennial seeds with sawdust at a ratio of 1:2 and spread 1.3 kg of that mixture at each site to overwinter (Landis, Wratten & Gurr, 2000; Fiedler & Landis, 2007a; Fiedler & Landis, 2007b). The perennial floral insectaries were mowed by the growers once per month to enhance establishment during the 2011 growing season.

### Quantifying pollinator assemblages and activity using video surveillance

A modified 4-channel security camera system (Q-see, model no. QSC26404, Anaheim, CA) was used to monitor pollinator activity within two female pumpkin flowers and two male pumpkin flowers within each pumpkin plot (total of 8 female and 8 male flowers observed per site) (Grieshop et al., 2012). Cameras recorded pollinator activity between 0600 h and 1200 h, at 16 frames per second with a playback pixel resolution of 352 × 240 (aspect ratio ∼1.222:1).

In both 2011–12, video surveillance was conducted once during peak bloom in late-July through August. We then omitted pumpkin sites 5, 10, and 13 in 2011 (*n* = 9 pumpkin plots sampled) due to a wet spring that resulted in an uncommonly late planting, and peak bloom period in September, which we felt was too late to accurately represent the pollinator community that focuses on the pumpkin flower resource pulse. In 2012, pumpkin sites 3p and 7p (*n* = 16 pumpkin sites sampled) could not be sampled due to heavy weed pressures that drastically reduced pumpkin bloom availability.

After collection, the video footage was transferred to portable hard-drives and stored until viewed on a computer. When a pollinator was observed crossing the plane made by the open corolla, the time of arrival and departure was recorded as a measure of the amount of time spent inside the flower. All pollinators were identified to the lowest taxonomic level possible given the resolution of the video.

### Measuring pollen deposition

In 2011 and 2012, we quantified the pollination service provided to each pumpkin site using pollen counts. In 2011 we examined cumulative pollen deposition across three lengths of the pollination window: 2 h (0600–0800 h), 4 h (0600–1000 h) and what we considered the full pollination window of 6 h (0600–1200 h). In 2012 we modified how we measured pollen deposition, collecting data for three individual subsets throughout the pollination window (0600–0800 h, 0800–1000 h, and 1000–1200 h), as well as across the entire period 0600–1200 h. One day prior to the collection of data, mature female flower buds that were at least 5 cm in length and turning deep yellow were located within each site, fitted with a mesh paint strainer bag (Reaves and Co. Durham, North Carolina, USA) as a pollinator excluder, and marked with a step-in poly post (Gempler’s, Madison, Wisconsin, USA). Three (2011) or six (2012) flowers were randomly assigned to each pollen deposition time treatment per pumpkin plot. Bags were left on flowers until the beginning of the treatment time upon which they were removed and pollinators were allowed to access flowers. If the number of flowers needed could not be found for each treatment on the morning of the experiment, we returned within seven days of the first attempt, and in comparable weather conditions to collect additional replicates.

### Pollen collection

We designed a simple and inexpensive procedure to collect pollen from stigmas in the field directly after cutting the flower from the pumpkin plant, based in part on the shake and rinse approach of Stanghellini, Schultheis & Ambrose (2002). We used an Aeropress espresso maker and the stock filter discs marked with a 1 × 1 cm grid (Aerobie, Inc., Palo Alto, California, USA) to sieve pollen grains from each collected stigma. Stigmas were placed individually in a 120 mL urine specimen cup with ∼44 mL of a dish soap and water solution (4 drops of dish soap per 2 L of water) and shook vigorously for 20 s. The solution was decanted into a separate cup and the stigma was washed a second time with 70% ethanol. The pollen solution was then poured into the Aeropress, and expunged. The inside of the Aeropress was washed with ethanol so that any pollen that was sticking to the sides was collected on the filter. The filter disc containing the pollen was allowed to dry, packaged individually in labeled petri dishes, and frozen until they were counted under a microscope. Pollen grains from six randomly selected full grid squares, and six partial grid squares were counted and the total pollen load on each filter disc was extrapolated.

### Quantifying landscape composition

We obtained aerial image mosaics of each county that contained a research site from the year 2010 (OGRIP, 2010) and uploaded them into ArcMap (version 9.3; ESRI, 2011) and QGIS (version 1.8.0; Quantum GIS Development Team, 2012) to digitize all land cover elements. We determined the area of each distinct landscape feature within 500, 1,000, and 1,500 m radius buffers around the geographic center of each site and ground verified them with a classification system including 22 habitat types. The 22 fine-grain cover types were combined into 7 coarse-grain habitat categories, and the percentages of each habitat type were aggregated as predictor variables within each landscape buffer for analysis (FIELD = percentage of annual field crops; GRASSLAND = percentage of perennial grassland, fallow fields, and pastureland; FORAGE = perennial alfalfa and oats; FRUITVEG = fruit and vegetable cropland; FOREST = woodlands and hedgerows; URBAN = impervious surfaces and buildings; TURF = mowed turfgrass). Total semi-natural habitat (FOREST and GRASSLAND) in each landscape ranged from 10.7–57.7% within a 1,500 m buffer.

### Statistical analyses

#### Visitation frequency

The frequency of total flower visits (fixed factor = bee species), male and female flower visitation (fixed factors = bee species and flower sex), and visitation length (fixed factors = bee species and flower sex) were examined for the three most abundant taxa visiting pumpkin flowers (*A. mellifera*, *Bombus* spp., and *P. pruinosa*) using generalized linear mixed models (glmmadmb function in the glmmADMB package version 0.7.4 in R version 3.0.0) with a Laplace maximum likelihood approximation that allowed for specification of a logistic link function, a negative binomial error distribution for visit frequency data, and a gamma distribution for visit duration data (R Development Core Team, 2013). We also examined how bee visitation to flowers varied by hour of the pollination window using a generalized linear mixed model with the fixed factors flower sex (male or female), bee species (*A. mellifera*, *Bombus* spp., or *P. pruinosa*) and time period (0600–0700, 0701–0800, 0801–0900, 0901–1000, 1001–1100, and 1101–1200 h) and site as a random factor. We used multiple comparisons procedures to contrast the fixed covariates within each model.

#### Pollen deposition

We modeled the number of pollen grains collected from stigmas of female flowers from three (2011: 2 h (0600–0800 h), 4 h (0600–1000 h) and 6 h (0600–1200 h) or four (2012, 0600–0800 h, 0800–1000 h, and 1000–1200 and 0600–1200 h) time periods using the glmmadmb mixed model function with a negative binomial distribution, and the general linear hypothesis test (glht) function from the multcomp package in R to test for significant differences between time periods.

#### Habitat management and landscape

To assess whether local habitat management or surrounding landscape composition influenced bee visitation frequency or pollination services we used partial least squares regression analysis (PLS). PLS allows for analysis of models with: (1) multiple response variables, (2) a large number of predictors which may be collinear, and (3) small samples sizes relative the number of possible predictor variables (Carrascal, Galvan & Gordo, 2009). As our landscape variables were proportions of buffer circles, many categories were highly correlated (Appendix S1). PLS reduces sets of predictor and response variables into a smaller set of latent factors.

For 2011, we examined the influence of 21 landscape variables (FOREST, GRASSLAND, FORAGE, FIELD, FRUITVEG, URBAN, AND TURF at 500 m, 1,000 m and 1,500 m radii surrounding each sampling site) on bee visitation frequency and pollen deposition (600–1200 h). In 2012, the influence of the three habitat management treatments (GRASS CONTROL, ALYSSUM and PERENNIAL) was also included in PLS models that examined bee visitation frequency (flower visitation by *A. mellifera*, *Bombus spp.*, and *P. pruinosa*), flower visitation by *Bombus* spp. only (examined as *Bombus* spp. represented 76.2% of flower visits in 2012), and pollen deposition (600–1200 h). In both years, visitation and pollen deposition were considered separately due to different timing of the experiments.

All predictor variables were centered to a mean of zero and scaled to a standard deviation of one, to give all variables equal weight. The number of factors to be extracted was determined by cross validation using a minimum predicted residual sum of squares (PRESS) as the stop condition. Explanatory variables with a Variable Importance in Projection (VIP) score of >0.8 for a given component were considered significant predictors for that component (SAS Institute Inc, 2011). For each analysis we interpreted up to the first two components (*t*_1_ and *t*_2_) and only those with a positive *Q*^2^ score. Correlation loading plots were used to explore the relationship between the predictor and response variables. These analyses were conducted using the PLS Module of XLSTAT (Addinsoft, Paris, France).

## Results

A total of 1,427 *A. mellifera* (47.7%), 606 *Bombus* spp. (20.2%), 898 *P. pruinosa* (30.0%), and 61 other pollinators (2%) were observed in male and female pumpkin flowers in 2011. In 2012 we observed 826 *A. mellifera* (10.5%), 6,023 *Bombus* spp. (76.2%), 964 *P. pruinosa* (12.2%), and 87 other pollinators (1.0%) visiting to pumpkin flowers. Taxa in the other pollinators category included *Melissodes bimaculata*, Halictidae, Andrenidae and Syrphidae. The number of flower visits by *A. mellifera* was significantly greater in 2011 than *Bombus* spp. (*z* = − 6.23, *P* < 0.001) or *P. pruinosa* (*z* = − 5.85, *P* < 0.001). The frequency of *Bombus* spp. and *P. pruinosa* visitation did not differ (*z* = 0.65, *P* = 0.783). In 2012, *Bombus* spp. were the most frequent visitor, compared to *A. mellifera* (*z* = 7.42, *P* < 0.001) and *P. pruinosa* (*z* = − 6.31, *P* < 0.001). There was no difference in the number of visits by *A. mellifera* and *P. pruinosa* (*z* = − 1.3, *P* = 0.382).

### Male versus female flower visitation

**Figure 2:**
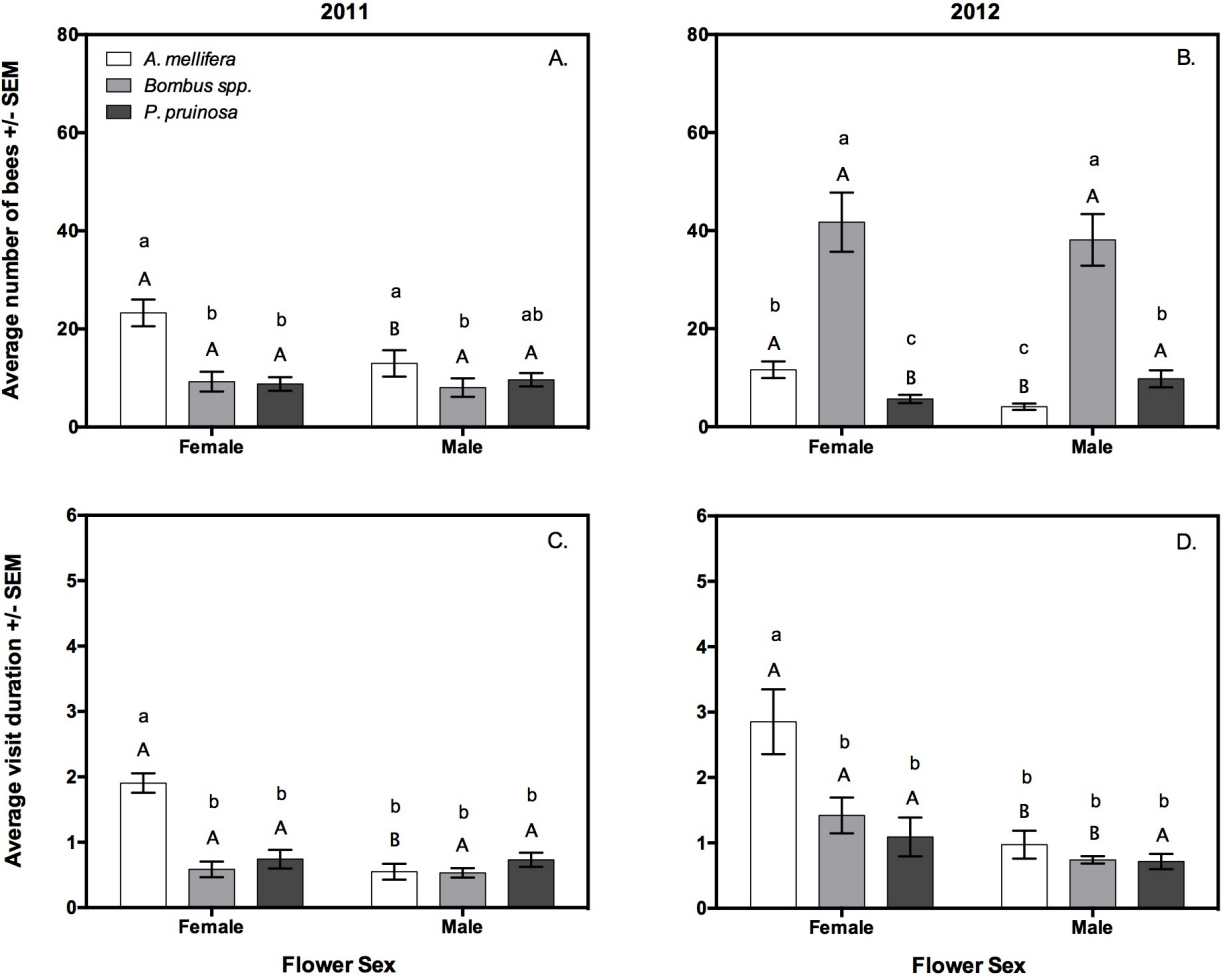
The average number of visits (A, B), and average visit duration in minutes of bees (C, D) to male and female flowers in 2011 (A, C) and 2012 (B, D), as observed by video cameras. Capital letters indicate significant differences within species across flower sex, while lower case letters indicate significant differences among species within a flower sex.

In 2011, *A. mellifera* visited female flowers more frequently than male flowers (*z* = − 3.26, *P* = 0.001), and spent more time in female flowers (*z* = − 8.1, *P* < 0.001) (Fig. 2). For *Bombus* spp., the number of visits to male and female flowers (*z* = − 0.74, *P* = 0.461) and the duration of each foraging bout (*z* = − 0.53, *P* = 0.594) did not differ (Fig. 2). Similarly, *P. pruinosa* visit frequency (*z* = 1.05, *P* = 0.295) and duration (*z* = 0.02, *P* = 0.983) did not vary among male and female flowers (Fig. 2).

In 2012, *A. mellifera* again visited female flowers more frequently (*z* = − 5.42, *P* < 0.001), and spent more time within them than male flowers (*z* = − 4.28, *P* < 0.001) (Fig. 2). *Bombus* spp. visitation frequency did not vary by flower sex, but bumble bees spent significantly more time in female flowers (*z* = − 3.24, *P* = 0.001) (Fig. 2). Squash bees visited male flowers more frequently (*z* = 2.48, *P* = 0.013), but individuals spent equal time in both flower sexes (Fig. 2).

### Variation among pollinators in male and female flower visitation

In 2011, *A. mellifera* visited male flowers more frequently than *Bombus* spp. (*z* = − 3.1, *P* = 0.005), but not *P. pruinosa* (*z* = − 2.04, *P* = 0.098). *Apis mellifera* also visited female flowers more frequently than *Bombus* spp. (*z* = − 4.7, *P* < 0.001) as well as *P. pruinosa* (*z* = − 5.06, *P* < 0.001) (Fig. 2). There was no difference in the number of times *Bombus* spp. and *P. pruinosa* visited male flowers (*z* = 0.91, *P* = 0.625) or female flowers (*z* = 0.03, *P* = 0.999) (Fig. 2). The duration of visits to male flowers did not differ between any bee species, but *A. mellifera* spent more time in female flowers than both *Bombus* spp. (*z* = − 5.28, *P* < 0.001), and *P. pruinosa* (*z* = − 4.56, *P* < 0.001) (Fig. 2).

In 2012, *Bombus* spp. visited male and female flowers more often than *A. mellifera* (*z* = 8.65, *P* < 0.001 in male and *z* = 4.48, *P* < 0.001 in female flowers), and *P. pruinosa* (*z* = − 4.14, *P* < 0.001 in male and *z* = − 5.66, *P* < 0.001 in female flowers) (Fig. 2). *Apis mellifera* visited female flowers more often (*z* = − 3.16, *P* = 0.004) and male flowers less often (*z* = 2.59, *P* = 0.024) than *P. pruinosa*. Honey bees also spent more time in female flowers per visit than *Bombus* spp. (*z* = 3.11, *P* = 0.005) or *P. pruinosa* (*z* = − 4.44, *P* < 0.001) (Fig. 2).

### Pollinator activity in flowers throughout the pollination window

**Figure 3:**
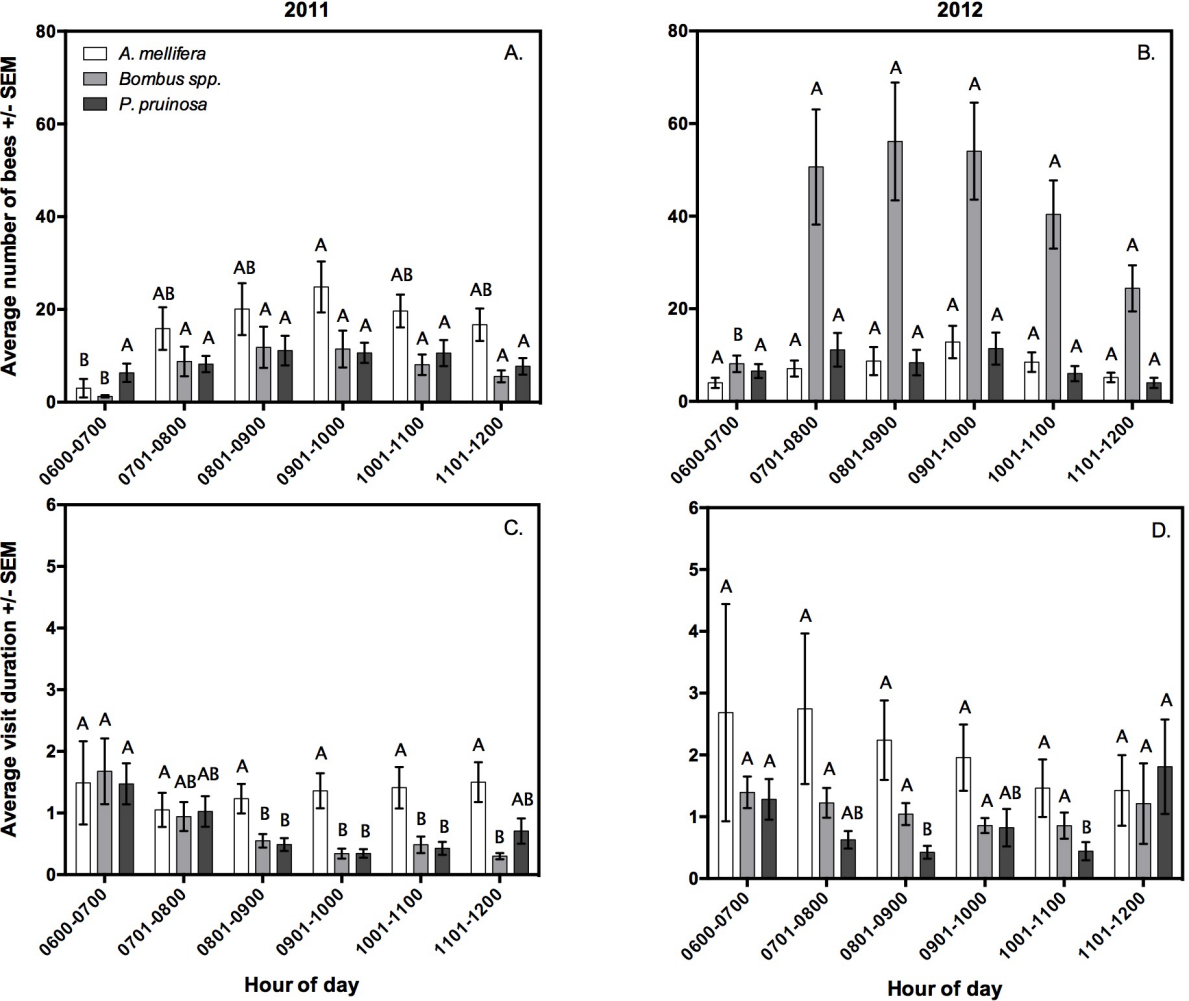
The average number of visits (A, B), and average visit duration in minutes by bees (C, D) between 0600 h and 1200 h in 2011 (A, C) and 2012 (B, D), as observed by video cameras. Letters indicate significant differences within species across hour.

In 2011, *A. mellifera* visitation to female flowers was relatively consistent across the pollination window, with a peak between 0901–1000 h wherein bees visited flowers more frequently than between 0600–0700 h (*z* = 3.07, *P* = 0.024) (Figs. 3A and 3C). The time *A. mellifera* spent inside flowers did not significantly differ by hour. In 2012, *A. mellifera* flower visitation frequency and duration did not vary by hour (Figs. 3B and 3D).

In 2011, *Bombus* spp. visitation frequency was significantly greater after 0700 h (0701–0.800 h: *z* = 3.44, *P* = 0.007, 0801–0900 h: *z* = 3.99, *P* < 0.001, 0901–1000 h: *z* = 4.30, *P* < 0.001, 1001–1100 h: *z* = 3.69, *P* = 0.003, 1101–1200 h: *z* = 3.67, *P* = 0.003 when compared to 0600–0700 h), yet individuals spent significantly fewer minutes inside flowers after 0700 h (0701–0800 h: *z* = − 3.42, *P* = 0.007, 0901–1000 h: *z* = − 4.64, *P* < 0.001, 1001–1100 h: *z* = − 3.73, *P* = 0.003, 1101–1200 h: *z* = − 4.83, *P* < 0.001 when compared to 0600–0700 h) (Figs. 3A and 3C). In 2012, *Bombus* spp. again visited flowers more frequently after 0700 h (0701–0800 h: *z* = 6.53, *P* < 0.001, 0801–0900 h: *z* = 7.09, *P* < 0.001, 0901–1000 h: *z* = 7.29, *P* < 0.001, 1001–1100 h: *z* = 6.42, *P* < 0.001, 1101–1200 h: *z* = 4.72, *P* < 0.001 when compared to 0600–0700 h). In 2012, *Bombus* spp. spent equal time in the flowers throughout the observation period (Figs. 3B and 3D).

In 2011, *P. pruinosa* visitation frequency did not vary by time of day, but individuals spent significantly more time in the flowers between 0600–0700 h than at times between 0801–1000 h (0801–900 h: *z* = − 3.84, *P* = 0.002, 0901–1000 h: *z* = − 4.84, *P* < 0.001, 1001–1100 h: *z* = − 4.22, *P* < 0.001 when compared to 0600–0700 h) (Figs. 3A and 3C). In 2012, *P. pruinosa* visitation frequency did not vary by time of day, but individuals spent significantly more minutes inside flowers before 0801 h (*z* = − 3.53, *P* < 0.01), and after 1101 h (*z* = 3.21, *P* < 0.01 when 1101–1200 h was compared to 0801–0900 h, and *z* = 3.31, *P* = 0.012 when 1101–1200 h was compared to 1001–1100 h) (Figs. 3B and 3D).

### Pollen deposition throughout the pollination window

**Figure 4:**
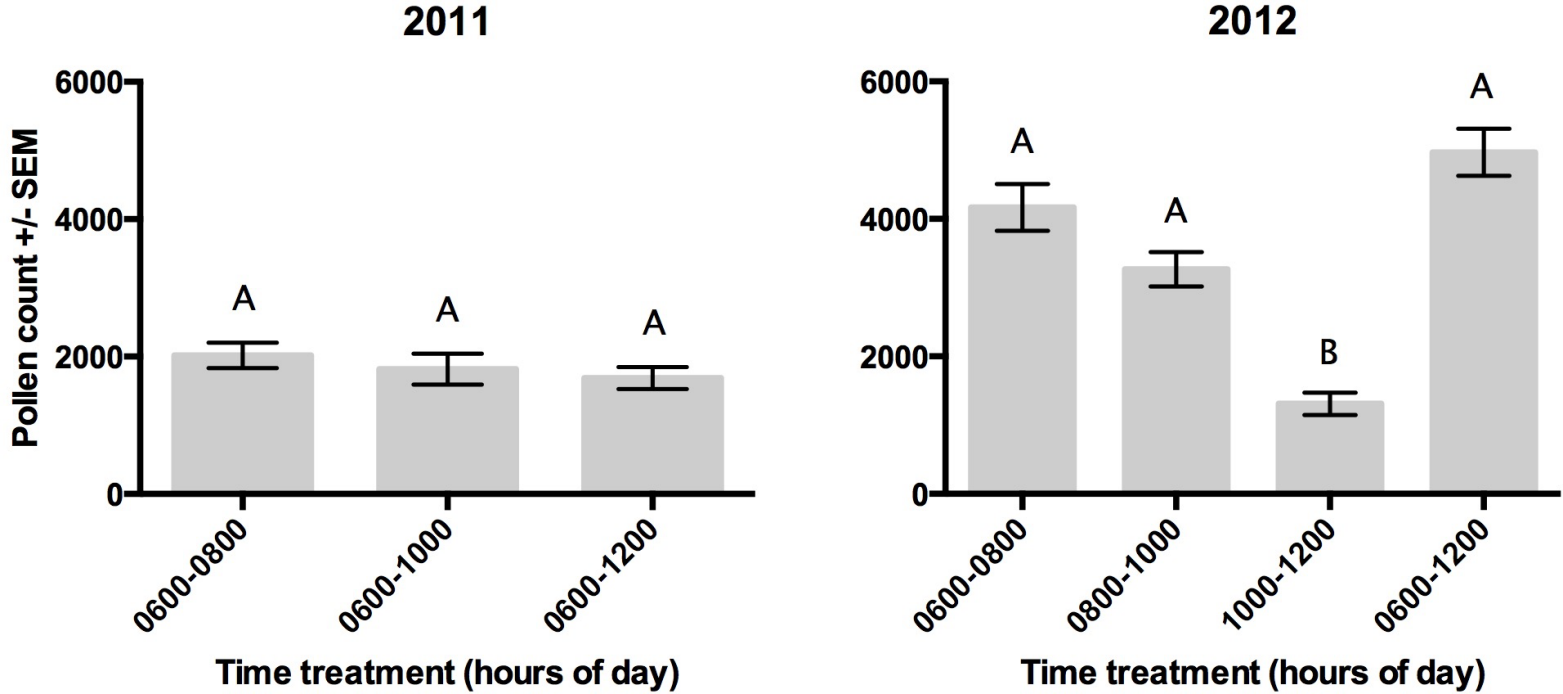
The average number of pollen grains deposited on pumpkin stigmas across all sites in 2011 was measured at three increasing time intervals: 0600–0800 h, 0600–1000 h, and 0600–1200 h. The average number of pollen grains across all sites in 2012 was measured at two-hour intervals and across the whole pollination window: 0600–0800 h, 0800–1000 h, 1000–1200 h and 0600–1200 h. Letters indicate significant differences among time periods within an observation year.

In 2011, we found no difference in pollen deposition between 0600–0800 h and 0600–1000 h (*z* = − 1.18, *P* = 0.45), between 0600–0800 h and 0600–1200 h (*z* = 0.26, *P* = 0.98) or between 0600–1000 h and 0600–1200 h (*z* = − 1.49, *P* = 0.29) (Fig. 4). In 2012, the amount of pollen deposited decreased over time, with significantly more pollen grains deposited in flowers between 0600–0800 h than between 1000–1200 h (*z* = − 3.98, *P* < 0.001). Total pollen transferred after a full morning (0600–1200 h) was not significantly different from the 0600–0800 h pollination period (*z* = 0.74, *P* = 0.871 (Fig. 4).

### Habitat management and landscape influences on visitation frequency and pollen deposition

**Figure 5:**
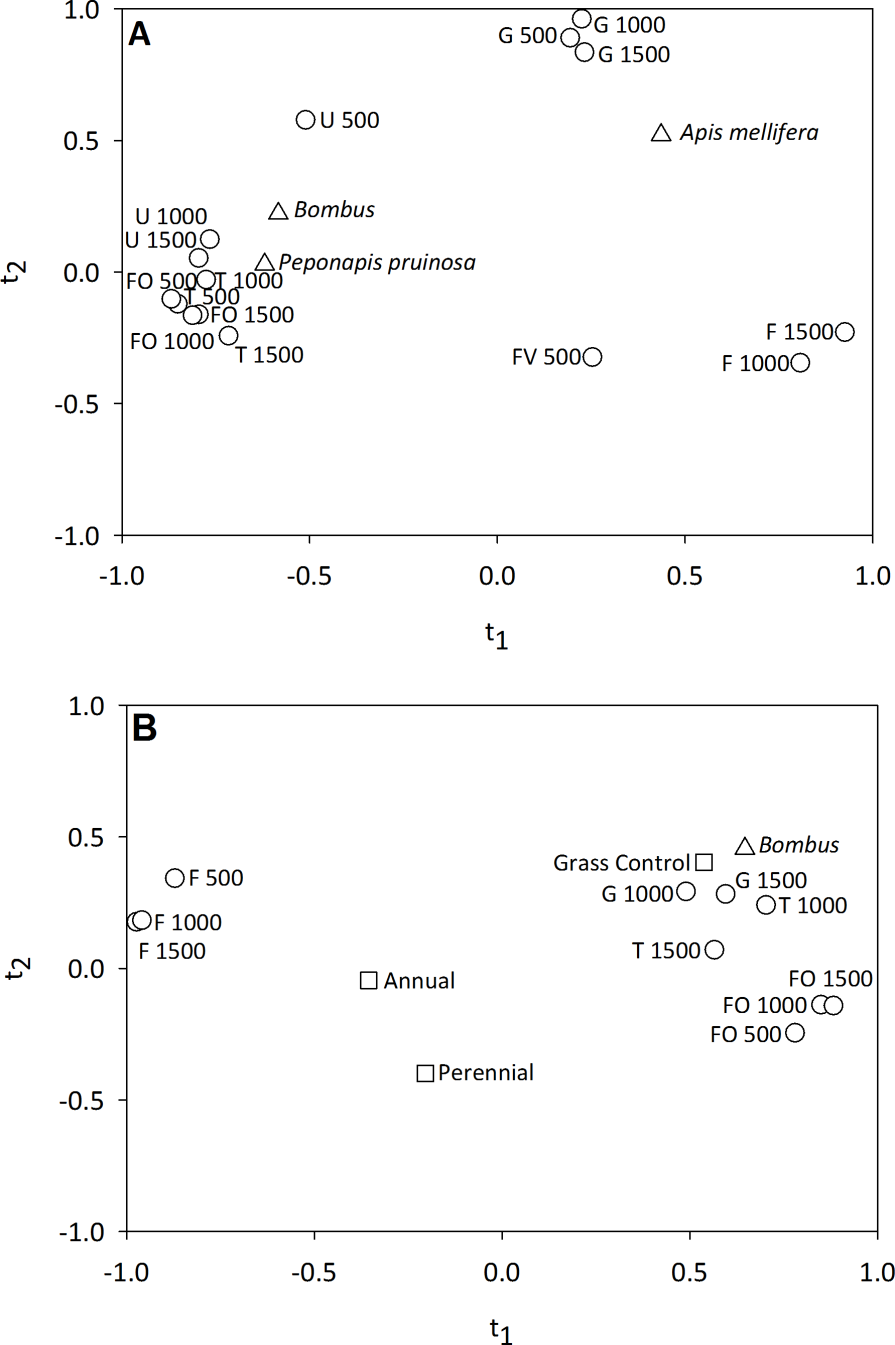
Correlation maps for the PLS regression of (A) pollinator visitation frequency and landscape variables in 2011 and (B) *Bombus* spp. visitation frequency and habitat management and landscape variables in 2012. Only landscape variables with a VIP score of >0.8 for a PLS component (*t*_1_ and *t*_2_) with a positive *Q*^2^ value are shown. In (B) habitat management variables are shown, but only Grass Control had a VIP score of >0.8, indicating that the addition of habitat management did not significantly influence *Bombus* spp. visitation to pumpkin flowers. Variable abbreviations as follows: Grassland (G), Forest (FO), Field Crops (F), Fruit and Vegetable Crops (FV), Urban (U) and Turf (T).

We found that bee visitation frequency was significantly related to landscape composition variables in 2011 and 2012. In 2011, both *t*_1_ and *t*_2_ had positive *Q*^2^ values. The *t*_1_ axis explained an average of 31% and *t*_2_ an additional 10% of the variation in visitation by *A. mellifera*, *Bombus* spp., and *P. pruinosa* (Table 3). For *t*_1_, 11 variables had a VIP score >0.8 (FOREST 500, 1000 and 1500; FIELD 1000, 1500; FRUITVEG 500, URBAN 1000, 1500 and TURF 500, 1000, 1500). *Bombus* spp. and *P. pruinosa* visitation were most strongly correlated with *t*_1_ (individual *R*^2^ = 0.34 and *R*^2^ = 0.39, respectively), and they visited pumpkin flowers in fields surrounded by urbanized areas and forest habitat more frequently than fields surrounded by a significant amount of corn, soybean and fruit and vegetable production (Fig. 5A). For *t*_2_, 15 variables had a VIP score >0.8 (FOREST 500, 1000, 1500; GRASSLAND 500, 1000, 1500; FIELD 1000, 1500; FRUITVEG 500; URBAN 500, 1000, 1500; AND TURF 500, 1000, 1500). *Apis mellifera* was most strongly correlated with *t*_2_ (individual *R*^2^ = 27.2%). We found that the number of honey bee visits to pumpkin flowers was greater in landscapes with significant amounts of grassland habitat and localized (500 km) urban habitat, and reduced in agricultural landscapes (Fig. 5A).

**Table 3:** Results of PLS regression analyses examining the influence of landscape variables (2011) and habitat management and landscape variables (2012) on pollinator visitation and pollen deposition. 2011 models included 21 landscape variables (FOREST, GRASSLAND, FORAGE, FIELD, FRUITVEG, URBAN AND TURF at 500 m, 1,000 m and 1,500 m radii surrounding each sampling site); 2012 models included the same landscape variables along with the categorical variable habitat management (GRASS CONTROL, ANNUAL OR PERENNIAL). For each model we report the *Q*^2^ (the proportion of the variance in the response variables that can be predicted by the model), the *R*^2^*Y* (the proportion of the variance in the response variable that is explained by the model) and *R*^2^*X* (the proportion of the variance in the matrix of predictor variables that is used in the model) for the first two model components (*t*_1_ and *t*_2_).

			*t*_1_			*t*_2_		
Year	PLS model	*X* variable(s)	*Q* ^2^	*R* ^2^ *Y*	*R* ^2^ *X*	*Q* ^2^	*R* ^2^ *Y*	*R* ^2^ *X*
2011	Visitation	*Apis mellifera*	0.17	0.31	0.41	0.12	0.39	0.56
		*Bombus* spp.						
		*Peponapis pruinosa*						
2012	Visitation	*Apis mellifera*	−0.12	0.12	0.32	−0.28	0.29	0.45
		*Bombus spp.*						
		*Peponapis pruinosa*						
2012	Bumblebee visitation	*Bombus* spp.	0.08	0.42	0.32	−0.58	0.63	0.42
2011	Deposition	Pollen grains	0.04	0.47	0.23	−0.44	0.56	0.55
2012	Deposition	Pollen grains	0.06	0.39	0.31	−0.54	0.47	0.5

In 2012, we found that neither habitat management nor landscape composition were significant predictors of bee visitation frequency when all three taxa were considered within a PLS model. Given the dominance of *Bombus* spp. in 2012 (76.2% of flower visits) a second model was examined considering only this group. For *Bombus* spp. alone we found that *t*_1_ explained 41.9% of the variation in bumble bee visitation to pumpkin flowers. A total of 11 variables had VIP scores of >0.8 on the *t*_1_ axis (FOREST 500, 1000, 1500; GRASSLAND 1000, 1500; FIELD 500, 1000, 1500, TURF 1000, 1500 and the habitat management variable GRASS CONTROL). We found that bumble bee visitation was highest in pumpkin fields lacking habitat management addition, embedded in landscapes dominated by semi-natural habitat and managed turf, and reduced in agricultural landscapes (Fig. 5B). For *Bombus* spp. alone, *t*_2_ had a negative *Q*^2^ value and was not evaluated.

**Figure 6:**
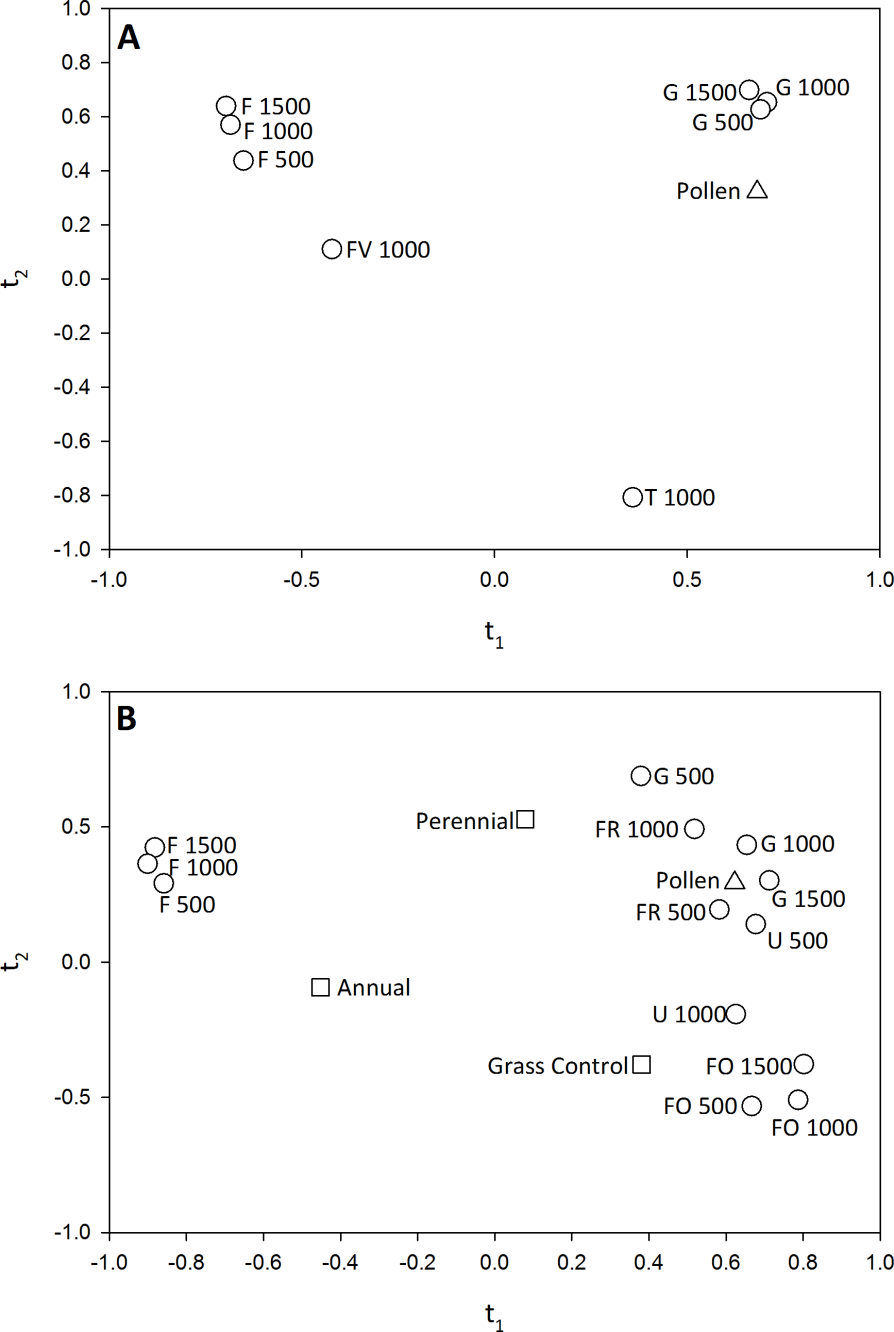
Correlation maps for the PLS regression of (A) pollen deposition and landscape variables in 2011 and (B) pollen deposition and habitat management and landscape variables in 2012. Only landscape variables with a VIP >0.8 for the PLS component *t*_1_ are shown. In (B) habitat management variables are shown, but none had a VIP score of >0.8, indicating that the addition of habitat management did not significantly influence pollen deposition. Variable abbreviations as follows: Grassland (G), Forest (FO), Forage (FR), Field Crops (F), Fruit and Vegetable Crops (FV), Urban (U) and Turf (T).

In both 2011 and 2012 pollen deposition was significantly related to landscape composition. In 2011, *t*_1_ explained 47% of the variation in pollen deposition; the *Q*^2^ value for *t*_2_ was negative and thus not examined. Eight variables had a VIP score of >0.8 along *t*_1_ (GRASSLAND 500, 1000, 1500; FIELD 500, 1000, 1500; FRUITVEG 1000 AND TURF 1000). Pollen deposition within pumpkin flowers was greater in fields surrounded by significant amounts of grassland habitat and mown turf and reduced in fields embedded in agriculturally-dominated landscapes (Fig. 6A). In 2012, we found that *t*_1_ explained 39% of the variation in pollen deposition. Again *t*_2_ had a negative *Q*^2^ value and was not evaluated. Thirteen variables had a VIP score of >0.8 along the *t*_1_ axis (GRASSLAND 500, 1000, 1500; FOREST 500, 1000, 1500; Forage 500, 1000; FIELD 500, 1000, 1500, and URBAN 500, 1000). Similarly to 2011, pollen deposition in pumpkin fields was greater within landscapes dominated by semi-natural and urban habitats and reduced in agriculturally-dominated landscapes (Fig. 6B). As with visitation frequency, we found that the addition of annual or perennial habitat management did not significantly influence pollen deposition.

## Discussion

Large seed set, successful maturation, and fruit weight are highly correlated with the number of pollinator visits to cucurbit flowers (Stanghellini, Ambrose & Schultheis, 1998; Garibaldi et al., 2013) and the amount of pollen transferred to female flowers per visit (Canto-Aguilar & Parra-Tabla, 2000; Winfree et al., 2007; Graças Vidal et al., 2010; Artz & Nault, 2011). Because of this close relationship, research on pollinators of cucurbits has often focused on the abundance of pollinators found inside flowers and the duration of their visitation (Tepedino, 1981; Cane, Minckley & Kervin, 2000; Shuler, Roulston & Farris, 2005; Julier & Roulston, 2009; Nicodemo, Nogueira Couto & De Jong, 2009; Barber, Adler & Bernardo, 2011; Artz, Hsu & Nault, 2011). Our work builds upon these studies using video surveillance to observe pollinator activity throughout the entire 6 h pumpkin pollination window, allowing for documentation of the composition of pollinator fauna visiting male and female flowers as well as their visitation frequency and duration. Further, we were able to measure how the local addition of habitat management as well as larger-scale landscape composition might influence the relationship between pollinator visitation and pollination service within this cropping system.

### Visitation frequency of pumpkin pollinators

We found significant variation in the dominant cucurbit pollinator across the two years of our investigation with *A. mellifera* representing 47.7% of pollinator visits in 2011-significantly more than either *Bombus* spp. or *P. pruinosa*. In 2012, *Bombus* spp. represented 76.2% of pollinator visits to flowers, far more than either *A. mellifera* or *P. pruinosa*, which had equivalent visitation frequencies. We saw a nearly a nine-fold increase in the number of bumble bee visits from 573 in 2011 to 5,069 in 2012. Although we do not know what factors contributed to this increase it is only partially explained by a greater number of sites sampled, from nine in 2011 to 16 in 2012. The winter of 2011–12 was among the warmest on record for Ohio, this combined with spring temperatures well above average may have increased survivorship of overwintering queens resulting in a greater number of foraging workers visiting pumpkin fields in 2012.

We aimed to determine if the three bee taxa contributing the majority of pumpkin pollination exhibited variation in how they partitioned their foraging activity among male and female flowers or their temporal use of these resources. Temporally, we found a high level of functional redundancy among this community of pollinators. We did not see much variation in the timing of flower visitation among species, with variation mainly found between the 0600–0700 h when bee activity tended to be lower than the remainder of the pollination window for all taxa. We expected squash bee to be active earlier in the pollination window than other bee species, based on Hurd, Linsley & Michelbacher (1974) who found *P. pruinosa* to be active 22–55 min before sunrise, and Tepedino (1981) who documented that most pollination provided by squash bee occurred before honey bees became prominent in the crop after 0800 h. However, we found the visitation frequency across the pollination window by this specialist to be relatively consistent with the other taxa. Later activity within flowers could be attributed to *P. pruinosa* males seeking flowers to shelter in for the afternoon and evening (Michelbacher, Smith & Hurd, 1964; Hurd, Linsley & Michelbacher, 1974).

We did find some differentiation in male versus female flower visitation among bee species. Honey bees visited female flowers more frequently than male flowers and spent more time in female flowers per visit. Bumble bees visited male and female flowers equally in both years, but like *A. mellifera* spent more time in female flowers in 2012. Similar to our results, Artz & Nault (2011) found that in New York pumpkin fields *A. mellifera* was more likely to visit female flowers and to spend more time in them. Female flowers produce significantly more nectar than male flowers; collecting nectar is likely to drive this foraging preference (Heinrich, 2004; Seeley, 2009). Unlike the social bees, squash bees visited male flowers more frequently in 2012. Tepedino (1981) also found that *P. pruinosa* visited more male flowers than female flowers. Similar to other soil and cavity-nesting solitary bees, female *P. pruinosa* rely more on pollen resources than nectar for solitary brood production.

### Pollination services in pumpkin fields

In both 2011 and 2012 we found that the majority of pollen deposition occurred within the first two hours (0600–0800 h) of observation. In fact, there was no difference between the pollen deposited during this two-hour period and the remainder of the pollination window (0600–1200 h) in either year. Graças Vidal et al. (2010) cite 1,500–2,000 pollen grains per flower as a requirement for complete pumpkin pollination. Based on this, the pumpkin plots included in our study received sufficient pollen deposition within just the first two hours of the pollination window.

We saw much higher pollen deposition in 2012 versus 2011, with an average of 4,188 (±294.49 SEM) pollen grains 2012 versus 2,017.59 (±252 SEM) in 2011 deposited between 0600–0800 h. The increase in pollen deposition in 2012 is likely attributable to the far greater visitation frequency by bumble bees. Bumble bees have been reported to be highly efficient pollinators, visiting 4–5 times more flowers per minute than honey bees (Fuchs & Müller, 2004) and carrying up to three times as many pollen grains per visit than *A. mellifera* or *P. pruinosa* (Artz, Hsu & Nault, 2011).

### Habitat management and pollination services

A key goal of this study was to determine how habitat management influenced the activity of both managed and wild pumpkin pollinators. We found no effect of either annual or perennial habitat management additions on bee visitation frequency or pollen deposition. Pumpkin fields received sufficient pollination services with or without the addition of habitat management.

Although we did not see an increase in ecosystem services delivered by the addition of plant resources, we do not want to convey that habitat management is without value in agricultural landscapes. With regard to our perennial plantings, a time lag may exist between the establishment of the habitat and any change in derived ecosystem services. Our perennial plantings were established in the fall of 2010, and sampled in their second growing season. It is very possible that their impact of on pollination and biocontrol services could change in subsequent years. Further, even if enhanced pest control and pollination are not achieved, perennial plantings have additional environmental benefits. They have been demonstrated to be important for conserving a diverse community of pollinators including those that tend to be most threatened by habitat loss and degradation (Haaland, Naisbit & Bersier, 2011; Wratten et al., 2012; Morandin & Kremen, 2013; Nicholls & Altieri, 2013; Balzan, Bocci & Moonen, 2014; Sardinas & Kremen, 2015; Wood et al., 2015). For example, Kremen & M’Gonigle (2015) found that habitat restoration within hedgerows enhanced the occurrences of native bee and syrphid fly, including taxa with more specialized nesting and foraging requirements and smaller pollinators with reduced mobility among patches.

Furthermore, recent evidence supports that in some agroecosystems these plantings can enhance pollination services. For example, Pereira et al. (2015) examined the utility of intercropping bell pepper with basil on pollination services and found that it increased the richness and abundance of bees visiting pepper flowers. Fruit produced in intercropped plots was also larger and contained more seeds than fruits produced on plots lacking basil plants (Pereira et al., 2015). Blaauw & Isaacs (2014) found that highbush blueberry growing adjacent perennial wildflower habitats exhibited enhanced fruit set, berry weight and mature seeds per berry. Honey bee visitation to blueberry flowers did not increase with wildflower habitat but wild bees and syrphid flies did (Blaauw & Isaacs, 2014). Similarly, Carvalheiro et al. (2012) found that mango orchards near plantings of perennial native plants had greater pollinator visitation and mango fruit production than orchards far from these additions.

### Landscape composition and pollinator visitation and pollination services

Landscape variables had a significant influence on bee visitation and pollen deposition. In 2011, bumble bees and squash bees were more abundant in pumpkin fields surrounded by forested and urbanized areas than in fields embedded in agricultural landscapes. In 2012, bumble bees were again more frequent pumpkin flower visitors in fields surrounded by managed turf and semi-natural habitats. We also found that pollen deposition in pumpkin fields was greater within landscapes dominated by semi-natural and urban habitats and reduced in agriculturally-dominated landscapes in 2011–12.

Several studies have found positive relationships between the abundance of semi-natural habitat, landscape heterogeneity, and wild bee abundance and pollination services in crop fields (Steffan-Dewenter et al., 2002; Ricketts et al., 2008; Klein et al., 2012; Kennedy et al., 2013; Andersson et al., 2014; Nayak et al., 2015). For example, Petersen & Nault (2014) used a conditional process modeling approach to illustrate that landscape diversity influenced the impact of bumble bees on pumpkin yield. Bumble bee visits to pumpkin flowers increased yield, but only in highly diverse landscapes (Petersen & Nault, 2014). Xie & An (2014) also found that bumble bee visitation to cucurbit flowers increased with the proportion of surrounding natural habitat, whereas honey bee visitation was unaffected by landscape. We found that landscape did influence honey bee foraging in 2011, with greater visitation by *A. mellifera* when fields were surrounded by grassland habitat and locally by urban areas. In 2012, however, like Xie & An (2014), we found no effect of landscape composition on honey bee visitation frequency.

In addition, to semi-natural habitat, we found a consistent positive correlation between wild bee visitation, pollen deposition, and the proportion of urban habitat in the surrounding landscape. Managed turf and gardens offer foraging and nesting resources for generalist pollinators like bumble bees (Hagen, Wikelski & Kissling, 2011; Samnegard, Persson & Smith, 2011; Gardiner, Burkman & Prajzner, 2013; Gunnarsson & Federsel, 2014; Parmentier et al., 2014). Additionally, many home and community gardens also produce cucurbit crops and support populations of squash bee. To date, it has not been demonstrated that these habitats serve as a source of either generalist or specialist pollinators to agricultural habitats, but our findings support additional investigation to quantify the value of urban habitats for pollinator conservation and pollination services.

### Conclusions

Habitat management seeks to mitigate the negative impacts of agricultural intensification on beneficial arthropods such as predators, parasitoids, and pollinators by providing alternative food and shelter resources (Landis, Wratten & Gurr, 2000; Zehnder et al., 2007). When habitat management practices are incorporated into a farmscape, larger scale landscape composition and heterogeneity structure the pool of beneficial species supplied to the floral insectary, which ultimately influences the arthropod-mediated ecosystem services they are able to support (Isaacs et al., 2009; Batáry et al., 2011; Concepción et al., 2012; Rodriguez-Saona, Blaauw & Isaacs, 2012). Tscharntke et al. (2012) introduced the Intermediate Landscape Complexity Hypothesis, which states that in highly heterogeneous landscapes (>20% non-crop habitats), stable populations of beneficial organisms already exist which limited the effect of local habitat management; and extremely simplified landscapes (<1% non-crop habitats) do not have enough supporting habitats for a substantial species pool to take advantage of local habitat amendments. As such, local habitat management is theoretically most useful to enhance arthropod-mediated ecosystem services within intermediately-complex landscapes. In 2012, when we evaluated the habitat management plantings only three landscapes fell into this intermediate landscape category, with all other sites having >20% non-crop habitat. To advance our understanding of the role of habitat management in provisioning ecosystem services, future work should explore whether we find a landscape threshold at which adding habitat resoruces on-farm alters the activity of insects that provide pollination or biocontrol services. Understanding these relationships would aid in the development of agri-environment schemes to enhance habitat for beneficial arthropods within the US where broad-scale implementataion of such plans lag behind those underway in the UK and continental Europe.

## Supplemental Information

10.7717/peerj.1342/supp-1 Supplemental Information 1 Raw data with keys to the legand

10.7717/peerj.1342/supp-2 Supplemental Information 2 Summary file of the 2011 pollen data

10.7717/peerj.1342/supp-3 Supplemental Information 3 Summary file of the 2011 pollinator data

10.7717/peerj.1342/supp-4 Supplemental Information 4 Summary file of 2012 pollen data

10.7717/peerj.1342/supp-5 Supplemental Information 5 Summary file of 2012 pollinator data

10.7717/peerj.1342/supp-6 Appendix S1 Appendix 1

## References

[ref-1] Aizen M, Harder L (2009). The truth about honeybees. New Scientist.

[ref-2] Allen-Wardell G, Bernhardt P, Bitner R, Burquez A, Buchmann S, Cane J, Cox PA, Dalton V, Feinsinger P, Ingram M, Inouye D, Jones CE, Kennedy K, Kevan P, Koopowitz H, Medellin R, Medellin-Morales S, Nabhan GP, Pavlik B, Tepedino V, Torchio P, Walker S (1998). The potential consequences of pollinator declines on the conservation of biodiversity and stability of food crop yields. Conservation Biology.

[ref-3] Andersson GKS, Ekroos J, Stjernman M, Rundlof M, Smith HG (2014). Effects of farming intensity, crop rotation and landscape heterogeneity on field bean pollination. Agriculture Ecosystems and Environment.

[ref-4] Artz DR, Hsu CL, Nault BA (2011). Influence of honey bee, *Apis mellifera*, hives and field size on foraging activity of native bee species in pumpkin fields. Environmental Entomology.

[ref-5] Artz DR, Nault BA (2011). Performance of *Apis mellifera*, *Bombus impatiens*, and *Peponapis pruinosa* (Hymenoptera: Apidae) as pollinators of pumpkin. Journal of Economic Entomology.

[ref-6] Baggen LR, Gurr GM (1998). The influence of food on *Copidosoma koehleri* (Hymenoptera: Encyrtidae), and the use of flowering plants as a habitat management tool to enhance biological control of potato moth, *Phthorimaea operculella* (Lepidoptera: Gelechiidae). Biological Control.

[ref-7] Balzan MV, Bocci G, Moonen AC (2014). Augmenting flower trait diversity in wildflower strips to optimize the conservation of arthropod functional groups for multiple agroecosystem services. Journal of Insect Conservation.

[ref-8] Barber NA, Adler LS, Bernardo HL (2011). Effects of above and below-ground herbivory on growth, pollination, and reproduction in cucumber. Oecologia.

[ref-9] Batáry P, Fischer J, Báldi A, Crist TO, Tscharntke T (2011). Does habitat heterogeneity increase farmland biodiversity?. Frontiers in Ecology and the Environment.

[ref-10] Blaauw BR, Isaacs R (2014). Flower plantings increase wild bee abundance and the pollination services provided to a pollination-dependent crop. Journal of Applied Ecology.

[ref-11] Blaker EA, Strange JP, James RR, Monroy FP, Cobb NS (2014). PCR reveals high prevalence of non/low sporulating *Nosema bombi* (microsporidia) infections in bumble bees (*Bombus*) in Northern Arizona. Journal of Invertebrate Pathology.

[ref-12] Bluthgen N, Klein A (2011). Functional complementarity and specialization: the role of biodiversity in plant–pollinator interactions. Basic and Applied Ecology.

[ref-13] Cameron SA, Lozier JD, Strange JP, Koch JB, Cordes N, Solter LF, Griswold TL (2011). Patterns of widespread decline in North American bumble bees. Proceedings of the National Academy of Sciences of the United States of America.

[ref-14] Campbell AJ, Biesmeijer JC, Varma V, Wäckers FL (2012). Realizing multiple ecosystem services based on the response of three beneficial insect groups to floral traits and trait diversity. Basic and Applied Ecology.

[ref-15] Cane JH, Minckley RL, Kervin LJ (2000). Sampling bees (Hymenoptera: Apiformes) for pollinator community studies: pitfalls of pan-trapping. Journal Kansas Entomological Society.

[ref-16] Canto-Aguilar MA, Parra-Tabla V (2000). Importance of conserving alternative pollinators: assessing the pollination efficiency of the squash bee, *Peponapis limitaris* in *Cucurbita moschata* (Cucurbitaceae). Journal of Insect Conservation.

[ref-17] Carrascal LM, Galvan I, Gordo O (2009). Partial least squares regression as an alternative to current regression methods used in ecology. Oikos.

[ref-18] Carvalheiro LG, Seymour CL, Nicolson SW, Veldtman R (2012). Creating patches of native flowers facilitates crop pollination in large agricultural fields: mango as a case study. Journal of Applied Ecology.

[ref-19] Concepción ED, Díaz M, Kleijn D, Báldi A, Batáry P, Clough Y, Gabriel D, Herzog F, Holzschuh A, Knop E, Marshall EJP, Tscharntke T, Verhulst J (2012). Interactive effects of landscape context constrain the effectiveness of local agri-environmental management. Journal of Applied Ecology.

[ref-20] Fiedler AK, Landis DA (2007a). Plant characteristics associated with natural enemy abundance at Michigan native plants. Environmental Entomology.

[ref-21] Fiedler AK, Landis DA (2007b). Attractiveness of Michigan native plants to arthropod natural enemies and herbivores. Environmental Entomology.

[ref-22] Fiedler A, Tuell J, Isaacs R, Landis D (2007). Attracting beneficial insects with native flowering plants.

[ref-23] Fuchs R, Müller M (2004). Pollination problems in styrian oil pumpkin plants: can bumblebees be an alternative to honeybees?. Phyton.

[ref-24] Fuerst MA, McMahon DP, Osborne JL, Paxton RJ, Brown MJF (2014). Disease associations between honeybees and bumble bees as a threat to wild pollinators. Nature.

[ref-25] Gardiner MM, Burkman CE, Prajzner SP (2013). The value of vacant land for arthropods and the ecosystem services they support in changing urban landscapes. Environmental Entomology.

[ref-26] Garibaldi LA, Carvalheiro LG, Leonhardt SD, Aizen MA, Blaauw BR, Isaacs R, Kuhlmann M, Kleijn D, Klein AM, Kremen C, Morandin L, Scheper J, Winfree R (2014). From research to action: enhancing crop yield through wild pollinators. Frontiers in Ecology and the Environment.

[ref-27] Garibaldi LA, Steffan-Dewenter I, Kremen C, Morales JM, Bommarco R, Cunningham SA, Carvalheiro LG, Chacoff NP, Dudenhöffer JH, Greenleaf SS, Holzschuh A, Isaacs R, Krewenka K, Mandelik Y, Mayfield MM, Morandin LA, Potts SG, Ricketts TH, Szentgyörgyi H, Viana BF, Westphal C, Winfree R, Klein AM (2011). Stability of pollination services decreases with isolation from natural areas despite honey bee visits. Ecology Letters.

[ref-28] Garibaldi LA, Steffan-Dewenter I, Winfree R, Aizen MA, Bommarco R, Cunningham SA, Kremen C, Carvalheiro LG, Harder LD, Afik O, Bartomerus I, Benjamin F, Boreux V, Cariveau D, Chacoff NP, Dundenhoffer JH, Freitas BM, Ghozoul J, Greenleaf S, Hipolito J, Holzschuh A, Howlett B, Isaacs R, Javerek SK, Kennedy CM, Krewenka KM, Krishnan S, Mandelik Y, Mayfield MM, Motzke I, Munyuli T, Nault BA, Otieno M, Petersen J, Pisanty G, Potts SG, Rader R, Recketts TH, Rundlof M, Seymour CL, Schuepp C, Szentgyorgyi H, Taki H, Tscharntke T, Vergara CH, Viana BG, Wagner TC, Westphal C, Williams N, Klein AM (2013). Wild pollinators enhance fruit set of crops regardless of honey bee abundance. Science.

[ref-29] Gonzalez-Varo JP, Biesmeijer JC, Bommarco R, Potts SG, Schweiger O, Smith HG, Steffan-Dewenter I, Szentgyoergyi H, Woyciechowski M, Vila M (2013). Combined effects of global change pressures on animal-mediated pollination. Trends in Ecology and Evolution.

[ref-30] Goulson D, Lye GC, Darvill B (2008). Decline and conservation of bumble bees. Annual Review of Entomology.

[ref-31] Goulson D, Nicholls E, Botias C, Rotheray EL (2015). Bee declines driven by combined stress from parasites, pesticides, and lack of flowers. Science.

[ref-32] Graças Vidal M, De Jong D, Wien HC, Morse RA (2010). Pollination and fruit set in pumpkin (*Cucurbita pepo*) by honey bees. Brazilian Journal of Botany.

[ref-33] Grieshop M, Werling B, Buehrer K, Perrone J, Isaacs R, Landis D (2012). Big brother is watching: studying insect predation in the age of digital surveillance. American Entomology.

[ref-34] Gunnarsson B, Federsel LM (2014). Bumble bees in the city: abundance, species richness and diversity in two urban habitats. Journal of Insect Conservation.

[ref-35] Haaland C, Naisbit RE, Bersier LF (2011). Sown wildflower strips for insect conservation: a review. Insect Conservation and Diversity.

[ref-36] Hagen M, Wikelski M, Kissling WD (2011). Space use of bumble bees (*Bombus* spp.) revealed by radio-tracking. PLoS ONE.

[ref-37] Heinrich B (2004). Bumble bee economics.

[ref-38] Hurd PD, Linsley EG, Michelbacher AE (1974). Ecology of the squash and gourd bee, *Peponapis pruinosa*, on cultivated cucurbits in California (Hymenoptera: Apoidea).

[ref-39] Hurd PD, Linsley EG, Whitaker TW (1971). Squash and gourd bees (*Peponapis*, *Xenoglossa*) and the origin of the cultivated cucurbita. Evolution.

[ref-40] Isaacs R, Tuell J, Fiedler A, Gardiner M, Landis D (2009). Maximizing arthropod-mediated ecosystem services in agricultural landscapes: the role of native plants. Frontiers in Ecology and the Environment.

[ref-41] Johanowicz D, Mitchell E (2000). Effects of sweet alyssum flowers on the longevity of the parasitoid wasps *Cotesia marginiventris* (Hymenoptera: Braconidae) and *Diadegma insulare* (Hymenoptera: Ichneumonidae). Florida Entomologist.

[ref-42] Julier HE, Roulston TH (2009). Wild bee abundance and pollination service in cultivated pumpkins: farm management, nesting behavior and landscape effects. Journal of Economic Entomology.

[ref-43] Kennedy CM, Lonsdorf E, Neel MC, Williams NM, Ricketts TH, Winfree R, Bommarco R, Brittain C, Burley AL, Cariveau D, Carvalheiro LG, Chacoff NP, Cunningham SA, Danforth BN, Dudenhoffer JH, Elle E, Gains HR, Garibaldi LA, Gratton C, Holzschuh A, Isaacs R, Javorek SK, Jha S, Klein AM, Krewenka K, Mandelik Y, Mayfield MM, Morandin L, Neame LA, Otieno M, Park M, Potts SG, Rundlof M, Saez A, Steffan-Dewenter I, Taki H, Viana BF, Westphal C, Wilson JK, Greenleaf SS, Kremen C (2013). A global quantitative synthesis of local and landscape effects on wild bee pollinators in agroecosystems. Ecology Letters.

[ref-44] Klein AM (2011). Plant–pollinator interactions in changing environments. Basic and Applied Ecology.

[ref-45] Klein AM, Brittain C, Hendrix SD, Thorp R, Williams N, Kremen C (2012). Wild pollination services to California almond rely on semi-natural habitat. Journal of Applied Ecology.

[ref-46] Klein AM, Vaissiere BE, Cane JH, Steffan-Dewenter I, Cunningham SA, Kremen C, Tscharntke T (2007). Importance of pollinators in changing landscapes for world crops. Proceedings of the Royal Society B.

[ref-47] Kremen C, M’Gonigle LK (2015). Small-scale restoration in intensive agricultural landscapes supports more specialized and less mobile pollinator species. Journal of Applied Ecology.

[ref-48] Kremen C, Williams NW, Thorp RW (2002). Crop pollination from native bees at risk from agricultural intensification. Pest Management Science.

[ref-49] Landis DA, Wratten SD, Gurr GM (2000). Habitat management to conserve natural enemies of arthropod pests in agriculture. Annual Review of Entomology.

[ref-50] Lee JC, Andow DA, Heimpel GE (2006). Influence of floral resources on sugar feeding and nutrient dynamics of a parasitoid in the field. Ecological Entomology.

[ref-51] Losey J, Vaughan M (2006). The economic value of ecological services provided by insects. BioScience.

[ref-52] McMahon DP, Fuerst MA, Caspar J, Theodorou P, Brown MJF, Paxton RJ (2015). A sting in the spit: widespread cross-infection of multiple RNA viruses across wild and managed bees. Journal of Animal Ecology.

[ref-53] Meeus I, Brown MJF, De Graff DC, Smagghe G (2011). Effects of invasive parasites on bumble bee declines. Conservation Biology.

[ref-54] Michelbacher A, Smith RF, Hurd P (1964). Bees are essential...pollination of squashes, gourds and pumpkins. California Agriculture.

[ref-55] Moran D, Grzes IM, Skorka P, Szentgyorgyi H, Laskowski R, Potts SG, Woyciechowski M (2012). Abundance and diversity of wild bees along gradients of heavy metal pollution. Journal of Applied Ecology.

[ref-56] Morandin LA, Kremen C (2013). Hedgerow restoration promotes pollinator populations and exports native bees and adjacent fields. Ecological Applications.

[ref-57] Morse RA, Calderone NW (2000). The value of honey bees as pollinators of US crops in 2000. Bee Culture.

[ref-58] National Agricultural Statistics Service (2013). Vegetables 2012 Summary.

[ref-59] Nayak GK, Roberts SPM, Garratt M, Breeze TD, Tscheulin T, Harrison-Cripps J, Vogiatzakis IN, Stripe MT, Potts SG (2015). Interactive effects of floral abundance and semi-natural habitats on pollinators in field beans (*Vicia faba*). Agriculture Ecosystems and Environment.

[ref-60] Nicholls CI, Altieri MA (2013). Plant biodiversity enhances bees and other insect pollinators in agroecosystems. A review. 2013. Agronomy for Sustainable Development.

[ref-61] Nicodemo D, Nogueira Couto RH, Malheiros EB, De Jong D (2009). Honey bee as an effective pollination agent of pumpkin. Scientia Agricola.

[ref-62] Park MG, Blitzer EJ, Gibbs J, Losey JE, Danforth BN (2015). Negative effects of pesticides on wild bee communities can be buffered by landscape context. Proceedings of the Royal Society B.

[ref-63] Parmentier L, Meeus I, Cheroutre L, Mommaerts V, Louwye S, Smagghe G (2014). Commercial bumble bee hives to assess an anthropogenic environment for pollinator support: a case study in the region of Ghent (Belgium). Environmental Monitoring and Assessment.

[ref-64] Pereira ALC, Taques TC, Valim JOS, Madureira AP, Campos WG (2015). The management of bee communities by intercropping with flowering basil (*Ocimum basilicum*) enhances pollination and yield of bell pepper (*Capsicum annuum*). Journal of Insect Conservation.

[ref-65] Petersen JD, Nault BA (2014). Landscape diversity moderates the effects of bee visitation frequency to flowers on crop production. Journal of Applied Ecology.

[ref-66] Pimentel D, Wilson C, McCullum C, Huang R, Dwen P, Flack J, Tran Q, Saltman T, Cliff B (1997). Economic and environmental benefits of biodiversity. BioScience.

[ref-67] Pontin D, Wade M, Kehril P, Wratten S (2005). Attractiveness of single and multiple species flower patches to beneficial insects in agroecosystems. Annals of Applied Biology.

[ref-68] Potts SG, Biesmeijer JC, Kremen C, Neumann P, Schweiger O, Kunin WE (2010a). Global pollinator declines: trends, impacts and drivers. Trends in Ecology and Evolution.

[ref-69] Potts SG, Roberts SPM, Dean R, Marris G, Brown MA, Jones R, Neumann P, Settele J (2010b). Declines of managed honeybees and beekeepers in Europe?. Journal of Apicultural Research.

[ref-70] Pywell R, Warman E, Hulmes L, Nuttall P, Sparks T, Critchley C, Sherwood A (2006). Effectiveness of new agri-environment schemes in providing foraging resources for bumble bees in intensively farmed landscapes. Biological Conservation.

[ref-71] Quantum GIS Development Team (2012). Quantum gis geographic information system (open source geospatial foundation project). http://qgis.org/en/site/.

[ref-72] R Development Core Team (2013). R: a language and environment for statistical computing.

[ref-73] Rader R, Reilly J, Bartomeus I, Winfree R (2013). Native bees buffer the negative impact of climate warming on honey bee pollination of watermelon crops. Global Change Biology.

[ref-74] Rands SA (2014). Landscape fragmentation and pollinator movement within agricultural environments: a modeling framework for exploring foraging and movement ecology. PeerJ.

[ref-75] Ricketts TH, Regetz J, Steffan-Dewenter I, Cunningham SA, Kremen C, Bogdanski A, Gemmill-Herren B, Greenleaf SS, Klein AM, Mayfield MM, Morandin LA, Ochieng A, Viana BF (2008). Landscape effects on crop pollination services: are there general patterns?. Ecology Letters.

[ref-76] Rodriguez-Saona C, Blaauw BR, Isaacs R, Larramendy ML, Soloneski S (2012). Manipulation of natural enemies in agroecosystems: habitat and semiochemicals for sustainable insect pest control. Integrated pest management and pest control—current and future tactics.

[ref-77] Rollin O, Bretagnolle V, Fortel L, Guilbaud L, Henry M (2015). Habitat, spatial and temporal drivers of diversity patterns in a wild bee assemblage. Biodiversity and Conservation.

[ref-78] Rundlof M, Andersson GKS, Bommarco R, Fries I, Hederstrom V, Herbertsson L, Jonsson O, Klatt BK, Pedersen TR, Yourstone J, Smith HG (2015). Seed coting with a neonicotinoid insecticide negatively affects wild bees. Nature.

[ref-79] Samnegard U, Persson AS, Smith HG (2011). Gardens benefit bees and enhance pollination in intensively managed farmland. Biological Conservation.

[ref-80] Sanchez-Bayo F, Goka K (2014). Pesticide residues and bees–a risk assessment. PLoS ONE.

[ref-81] Sardinas HS, Kremen C (2015). Pollination services from field-scale agricultural diversification may be context dependent. Agriculture Ecosystems and Environment.

[ref-82] SAS Institute Inc (2011). The PLS procedure. SAS/STAT^®^ 9.3 user’s guide.

[ref-83] Scheper J, Remmer M, Van Kats R, Ozinga WA, Van der Linden GTJ, Schaminee JHJ, Siepel H, Kleijn D (2014). Museum specimens reveal loss of pollen host plants as key factor driving wild bee decline in The Netherlands. 2014. Proceedings of the National Academy of Sciences of the United States of America.

[ref-84] Seeley TD (2009). The wisdom of the hive: the social physiology of honey bee colonies.

[ref-85] Shackelford G, Steward PR, Benton TG, Kunin WE, Potts SG, Biesmeijer JC, Sait SM (2013). Comparison of pollinator and natural enemies—a meta-analysis of landscape and local effects on abundance and richness in crops. Biological Reviews.

[ref-86] Shuler RE, Roulston TH, Farris GE (2005). Farming practices influence wild pollinator populations on squash and pumpkin. Journal of Economic Entomology.

[ref-87] Southwick E, Southwick L (1992). Estimating the economic value of honey bees (Hymenoptera: Apidae) as agricultural pollinators in the United States. Journal of Economic Entomology.

[ref-88] Stanghellini MS, Ambrose JT, Schultheis JR (1998). Using commercial bumble bee colonies as backup pollinators for honey bees to produce cucumbers and watermelons. HortTechnology.

[ref-89] Stanghellini MS, Schultheis JR, Ambrose JT (2002). Pollen mobilization in selected Cucurbitaceae and the putative effects of pollinator abundance on pollen depletion rates. Journal of the American Society of Horticultural Science.

[ref-90] Steffan-Dewenter I, Munzenberg U, Burger C, Thies C, Tscharntke T (2002). Scale-dependent effects of landscape context on three pollinator guilds. Ecology.

[ref-91] Tepedino VJ (1981). The pollination efficiency of the squash bee (*Peponapis pruinosa*) and the honey bees (*Apis mellifera*) on summer squash (*Cucurbita pepo*). Journal of the Kansas Entomological Society.

[ref-92] Tscharntke T, Tylianakis JM, Rand TA, Didham RK, Fahrig L, Batáry P, Bengtsson J, Clough Y, Crist TO, Dormann CF, Ewers RM, Frund J, Holt RD, Holzschuh A, Klein AM, Kleijn D, Kremen C, Landis DA, Laurance W, Lindenmayer D, Scherber C, Sodhi N, Steffan-Dewenter I, Thies C, Van der Putten WH, Westphal C (2012). Landscape moderation of biodiversity patterns and processes—eight hypotheses. Biological Reviews.

[ref-93] Tuell JK, Fiedler AK, Landis D, Isaacs R (2008). Visitation by wild and managed bees (Hymenoptera: Apoidea) to eastern US native plants for use in conservation programs. Environmental Entomology.

[ref-94] Vanbergen AJ, Baude M, Biesmeijer JC, Britton NF, Brown MJF, Brown M, Bryden J, Budge GE, Bull JC, Carvell C, Challinor AJ, Connolly CN, Evans DJ, Feil EJ, Garratt MP, Greco MK, Heard MS, Jansen VAA, Keeling MJ, Kunis WE, Marris GC, Memmott J, Murray JT, Nicolson SW, Osborne JL, Paxton RJ, Pirk CWW, Polce C, Potts SG, Priest NK, Raine NE, Roberts S, Ryabov EV, Shafir S, Shirley MDF, Simpson SJ, Stevenson PC, Stone GN, Termansen M, Wright GA (2013). Threats to an ecosystem service: pressures on pollinators. Frontiers in Ecology and the Environment.

[ref-95] Wien H (1997). The cucurbits: cucumber, melon, squash and pumpkin. The physiology of vegetable crops.

[ref-96] Winfree R, Williams NM, Dushoff J, Kremen C (2007). Native bees provide insurance against ongoing honey bee losses. Ecology Letters.

[ref-97] Wood TJ, Holland JM, Hughes WOH, Goulson D (2015). Targeted agri-environment schemes significantly improve the population size of common farmland bumble bee species. Molecular Ecology.

[ref-98] Wratten SD, Gillespie M, Decourtye A, Mader E, Desnuex N (2012). Pollinator habitat enhancement: benefits to other ecosystem services. Agriculture Ecosystems and Environment.

[ref-99] Xie Z, An J (2014). The effects of landscape on bumble bees to ensure crop pollination in the highland agricultural ecosystems in China. Journal of Applied Entomology.

[ref-100] Zehnder G, Gurr GM, Kühne S, Wade MR, Wratten SD, Wyss E (2007). Arthropod pest management in organic crops. Annual Review of Entomology.

[ref-101] Zhang W, Ricketts TH, Kremen C, Carney K, Swinton SM (2007). Ecosystem services and dis-services to agriculture. Ecological Economics.

