# Supplementary material for: Use of video surveillance to measure the influences of habitat management and landscape composition on pollinator visitation and pollen deposition in pumpkin (Cucurbita pepo) agroecosystems
Source: PeerJ. 2015 Nov 5;3:e1342. doi: 10.7717/peerj.1342 (PMC4647596; doi:10.7717/peerj.1342)
Supplement: Appendix S1 [file peerj-03-1342-s006.docx]

Appendix 1: Correlations among landscape variables at 500, 1000 and 1500 m in 2011 and 2012.
